# Role of the TSPO–NOX4 axis in angiogenesis in glioblastoma

**DOI:** 10.3389/fphar.2022.1001588

**Published:** 2022-10-07

**Authors:** Hongxiang Jiang, Fei Li, Linzhi Cai, Qianxue Chen

**Affiliations:** ^1^ Department of Neurosurgery, Renmin Hospital of Wuhan University, Wuhan, China; ^2^ Central Laboratory, Renmin Hospital of Wuhan University, Wuhan, China

**Keywords:** NADPH oxidase 4, translocator protein, angiogenesis, glioblastoma, reactive oxygen species

## Abstract

**Objective:** Angiogenesis is a pathological feature of glioblastoma. Nicotinamide adenine dinucleotide phosphate oxidase 4 (*NOX4*) is a vital source of reactive oxygen species (ROS) related to angiogenesis. However, signaling pathways correlated with the isoform oxidase are unknown. The aim of this study was to elucidate the detailed mechanism of the role of *NOX4* in angiogenesis in glioblastoma.

**Methods:** Public datasets were searched for studies on immunohistochemistry and western blotting to evaluate *NOX4* expression in glioma. The location of *NOX4* expression was detected by immunofluorescence. We conducted conditional deletion of the translocator protein (TSPO) targeting the protein with the synthetic ligand XBD173 in the glioblastoma mouse model. *NOX4* downregulation was conducted with the NOX4 inhibitor GLX351322, and ROS production and angiogenesis were detected in glioma tissues.

**Results:** Clinical samples and public datasets showed that *NOX4* was upregulated and associated with the prognosis. *NOX4* is mainly expressed in endothelial cells of glioblastoma. Both TSPO and NOX4 promoted angiogenesis in an ROS-dependent manner, suggesting that TSPO triggered ROS production in glioblastoma *via* NOX4.

**Conclusion:** These results showed that TSPO is an upstream target of NOX4-derived mitochondrial ROS, which is indispensable for NOX4-derived mitochondrial ROS-induced angiogenesis in glioblastoma. TSPO–NOX4 signaling could serve as a molecular target for therapeutic strategies for glioblastoma.

## Introduction

Glioma, a common primary tumor of the central nervous system, has various heterogeneous subregions (Bakas et al., 2017). High-grade gliomas, such as glioblastoma, have a poor prognosis and often lead to death. Despite research advancements, the treatment is limited (Gusyatiner and Hegi, 2018). Glioblastoma could cause pathologic changes in angiogenesis, thereby accelerating tumor progression, making the treatment more difficult and increasing tumor aggressiveness (Kane, 2019). Owing to the special intratumoral heterogeneity and complex vascularization processes, various types of tumor cells regulate tumor-associated blood vessels in glioma through multiple molecular mechanisms. The tumor microenvironment consists of tumor cells and tumor-associated stroma, which could generate several types of molecular mediators to moderate angiogenesis in glioma.

High tumor microvascular density is a marker of glioblastoma and indicates a poor prognosis (Fan et al., 2019). To date, the exact regulation mechanism of angiogenesis-related factors has not been fully understood. Understanding this mechanism could yield effective treatment targets, particularly for drug intervention. Currently, altering metabolic gene expressions in tumors is the ideal therapeutic target for tumors (Liu et al., 2021). Oxidative stress plays a crucial role in cancer development and progression. Under physiological conditions, increased reactive oxygen species (ROS) production plays a key signaling function (Moloney and Cotter, 2018). Nicotinamide adenine dinucleotide phosphate oxidase 4 (NOX4) is a NOX family isoform correlated with ROS production and various biological functions in the development of cancer (Moloney and Cotter, 2018). NOX4-induced ROS production and increased *Nox4* expression play key roles in glioma (Wu et al., 2017). However, mechanisms underlying *Nox4*-derived ROS levels in glioblastoma are largely unknown.

Wolfe et al. (Wolf et al., 2020) showed that the translocator protein (TSPO) is a regulatory node for microglial functions in angiogenesis carried out in a NOX1-dependent manner. Since TSPO is enriched in glioma, it may be a diagnostic biomarker (Su et al., 2015). Positron emission tomography (PET) with radio-labelled TSPO ligands, such as [^11^C]PK11195, shows good abilities to monitor and quantify tumor progression (Su et al., 2013). Nevertheless, the function of TSPO in glioma or its potential molecular mechanism has not been fully detailed. The aim of this study was to perform a complete mechanistic analysis of NOX4-mediated TSPO-induced ROS elevation in angiogenesis in glioblastoma. The results could serve as a basis for exploring therapeutic targets based on the dual inhibition of NOX4 and TSPO in glioblastoma.

## Materials and methods

### Clinical samples

We obtained glioma tissues from the Department of Neurosurgery, Renmin Hospital, Wuhan University, from January 2018 to July 2021. Immunohistochemical staining was performed for 17 glioblastoma, 15 grade III malignant, 19 grade II malignant, 23 grade I malignant, and 11 healthy tumor-adjacent tissues, stored at −80°C. Supplementary Table S1 shows clinical data of all patients. Preoperatively, chemotherapy or radiotherapy was not performed in any patient. All patients provided written informed consent. The research protocol was approved by the ethics committee of Renmin Hospital, Wuhan University (approved number: WDRY2022-KS002).

### Cell culture

Murine Gl261 glioma cells were derived from the Tumor Cell Repository of the National Cancer Institute, Frederick, United States of America. They were cultured under adherent conditions in Dulbecco’s Modified Eagle Medium, containing 100 μg/ml penicillin, 100 μg/ml streptomycin, and 10% fetal bovine serum, at 37°C in a humidified atmosphere under 95% O_2_ and 5% CO_2_.

### Tumor inoculation

C57Bl/6J mice were purchased from Shulaibao Biotechnology Co., Ltd. Animal feeding and experimental protocols of this study conformed to the guidelines set by the Animal Ethics Committee of Renmin Hospital, Wuhan University. Gl261 glioma cells growing in the logarithmic phase were prepared. They were resuspended in phosphate-buffered saline (PBS) at 1×10^6^ cells/100 μL and then injected subcutaneously into the brains of 6-week-old C57Bl/6J mice. A layer of moisturizing cream covered their corneas. The scalp was disinfected with 10% potassium iodide solution, and a median incision was made on it with a scalpel. The mice were then fixed on a stereotaxic frame. A 23-G needle was used to drill holes in the skull at 1.5-mm puncture points in front and on the right side of the anterior fontanelle. Further, 1 µL PBS, containing 1 × 10^5^ Gl261 glioblastoma cells, was slowly injected into the brain of the mouse at a depth of 3 mm, using a 22-G Hamilton syringe, over 2 min. Finally, the syringe was pulled at a rate of 1 mm/min, and the skin was sutured. In mouse cells, the tumor grew for 3 weeks.

### Animal experiments

For the *in vivo* treatment, C57Bl/6J mice were randomly divided into treatment and control groups. *In vivo* experiments were performed to explore the role of NOX4 in tumorigenesis in an intracranial xenograft model. We divided all intracranial xenograft mice into three groups: treatment group 1, provided with 3.8 mg/day/kg body weight of GLX351322 (*NOX4* inhibitor, reducing *NOX4* expression) in normal saline served as drinking water every day for 2 weeks; treatment group 2 provided with GLX351322 in normal saline served as drinking water every day for 1 week; and control group, provided with no *NOX4* inhibitor. Each group included seven C57Bl/6J mice (Figure 2A). The intraperitoneal or gastric administration was initiated on day 7. Phenylpurine XBD173 (AC-5216, EMAPuniL) was obtained by custom-synthesis from APAC Pharmaceuticals. One week after tumor inoculation, the mice were injected intraperitoneally with XBD173 at 10 mg/kg, dissolved in dimethyl sulfoxide as carrier control. Each group included seven C57Bl/6J mice. After tumor inoculation, the C57Bl/6J status was observed daily. The dynamic weight change of each C57Bl/6J mouse was recorded weekly. To analyze survival, the mice were regularly monitored and sacrificed when they showed notable weight loss (over 20% of body weight) and/or significant neurological symptoms. The entire brain of sacrificed mice were wrapped in paraffin and preserved for further analyses. The aforementioned animal experiments were approved by the Institutional Animal Care and Use Committee of Renmin Hospital, Wuhan University.

### Mouse brain tissue preparation

Mouse brains were collected and then fixed in 4% sucrose for 24 h at 4°C and dehydrated in 30% sucrose at 4°C for ≥24 h. Subsequently, brain tissue was placed in a frozen matrix and slowly frozen to −80°C in isopentane. Tissue samples were obtained in 40-µm horizontal sections by sectioning horizontal slides. Finally, for protection from light, floating sections were stored at −20°C with a cryoprotective agent.

### Immunohistochemistry

The tissues were fixed with 4% paraformaldehyde, embedded in paraffin, and sectioned. After hydration, they were treated with 3% H_2_O_2_ for 10 min, blocked with 1% bovine serum albumin for 1 h, and incubated overnight with the anti-NOX4 primary antibody followed by the horseradish peroxidase-linked secondary antibody (Servicebio, China). Subsequently, they were stained with 3, 3′-diaminobenzidine and hematoxylin. Images were captured using an Olympus BX51 microscope (Olympus). The distribution and intensity of NOX4 staining (0 = negative; 1 = weak; 2 = moderate; 3 = strong; 4 = strong and widespread) were estimated by semi-quantitative scoring.

### Fluorescent immunohistochemistry and confocal microscopy

On day 1, the floating slices were cleaned thrice with PBS with 0.1% Tween-20 (PBST), for 5 min each time. Dual Endogenous Enzyme Block Dako was used at room temperature (25°C ± 5°C) for 10 min to block endogenous peroxidase, followed by three rinses with PBST. The sections were sealed for 1 h in PBS or a protein blocking reagent in the presence of 5% normal donkey serum and 0.3% Triton-X. Finally, they were incubated overnight at 4°C with the primary antibody diluted in the Dako antibody thinner. On day 2, the sections were cleaned and incubated with secondary antibodies diluted with the Dako Antibody Diluent for 2 h at room temperature (25°C ± 5°C) before coupling with fluorophore-conjugated streptavidin. Two caps were incubated together at room temperature (25°C ± 5°C). Nuclei were stained with 2 μg/ml 4′,6-diamidino-2-phenylindole. At the end of the experiment, the sections were placed on glass slides and allowed to air-dry for 10 min. Tissues were fixed in a fluorescent mounting medium and covered. At the Bioimaging Core Facility, confocal microscopy was performed using the Leica SP8X WLL microscope equipped with the 405-nm (470–670-nm) WLL2 laser and an acousto-optic beam splitter. Images were acquired using a 63 × 1.4 objective for visualization of single-cell and subcellular structures, with an image pixel size of 80 nm. To avoid leakage, the experiments were recorded sequentially. For all experiments, imaging was performed using the appropriate settings based on normalized negative and positive groups.

### Western blotting

The cells were washed with PBS at a low temperature (15°C ± 5°C) and lysed with radioimmunoprecipitation assay. The protein level in the supernatant was determined by the bicinchoninic acid protein assay. Further, 5% sodium dodecyl sulfate–polyacrylamide gel electrophoresis (SDS-PAGE) was added into lysates, followed by boiling at 98°C for 10 min. Total protein was loaded onto SDS-PAGE and electro-transferred on 0.22-μM polyvinylidene difluoride membranes, which were incubated with anti-GAPDH, anti-NOX4, CD31, and anti-TSPO primary antibodies overnight and secondary antibodies for 1 h at 25°C. Finally, the fixed western chemiluminescent horseradish peroxidase substrate (Micropores) was used to visualize protein bands.

### ROS level measurement

ROS levels were measured after XBD173 and GLX351322 treatments using the Reactive Oxygen Species Assay Kit and transfection with plasmids, following the manufacturer’s protocol. At 525 nm, cell absorbance was measured using a spectrophotometer.

### RNA extraction and quantitative real-time PCR

RNA extraction of related genes from tissues using TRIzol reagent (Invitrogen, Carlsbad, CA, United States). The RNA was then tested for purity as well as concentration. And a reverse transcription kit was applied in the convert of whole samples to cDNA. Performing qRT-PCR by employing SYBR Green (Thermo Fisher Scientific) system. GraphPad Prism 6.0. Was used to assess statistics. The results from the experimental and control groups were compared using the relative Ct technique, with GAPDH serving as an internal reference.

### Statistical analysis

Statistical analyses were performed using SPSS 19.0 and GraphPad Prism 6.0. *In vitro* experimental data are expressed as mean ± standard deviation. The one-way analysis of variance and the *t*-test were performed for between-group discrepancies. Each experiment was repeated in triplicate. A *p*-value < 0.05 was considered to indicate statistical significance.

## Results

### NOX4 overexpression and association with the prognosis of glioblastoma

**FIGURE 1:**
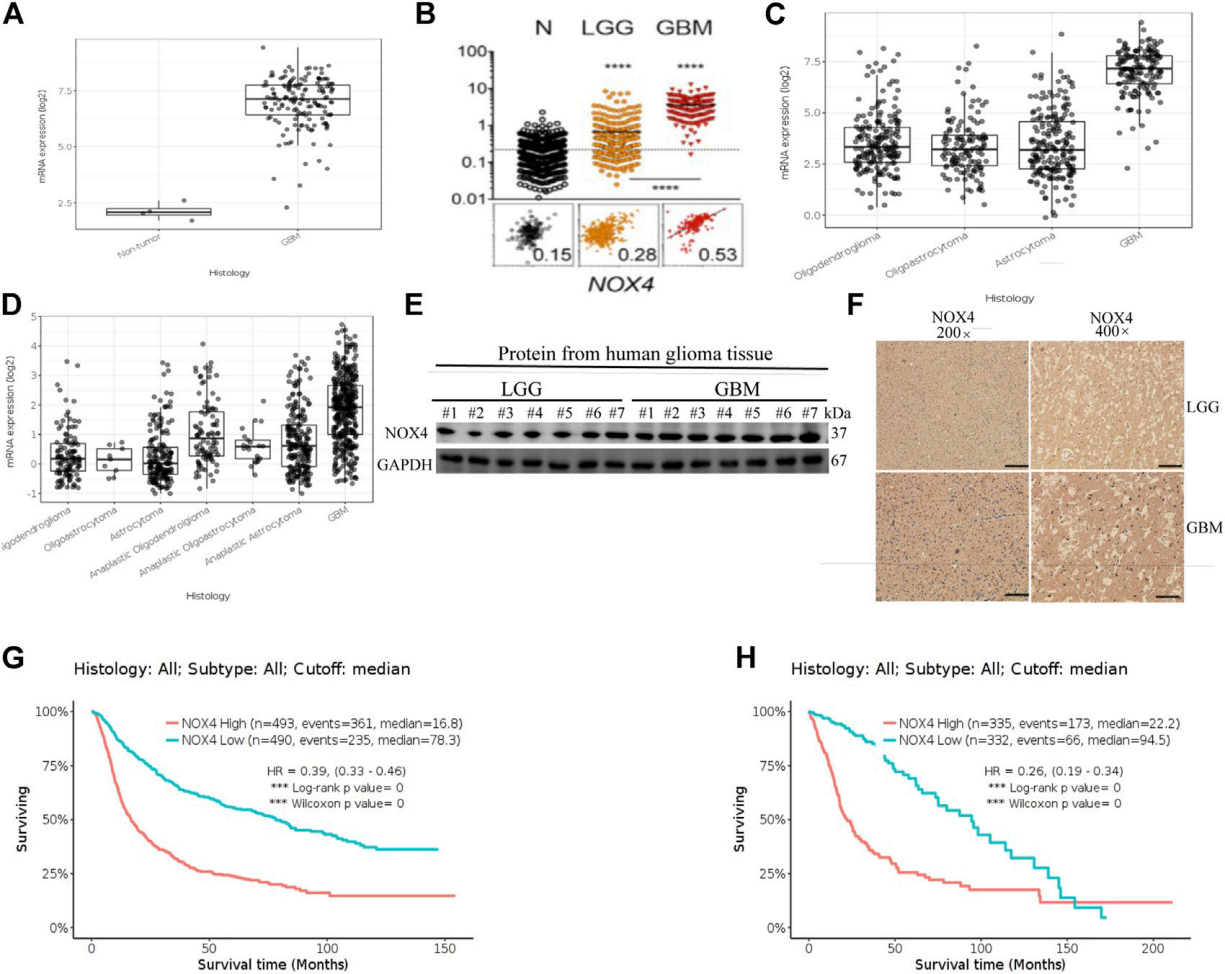
NOX4 is upregulated and associates with prognosis in glioma. **(A,B)** NOX4 mRNA expression in non-tumor tissue, low grade glioma (LGG) and high grade glioma (HGG) in TCGA datasets; **(C,D)** NOX4 mRNA expression in different histology in TCGA datasets and CGGA datasets; **(E)** Western blot was performed to compared FTL expression in LGG and GBM, LGG, n = 7; GBM, n = 7,GAPDH used as loading control; **(F)** Representative images of IHC staining of NOX4 in glioma tissues; **(G,H)** Kaplan-Meier survival analysis for NOX4 expression in all glioma patients in TCGA datasets and CGGA datasets. The median value of NOX4 levels was set as the cut-off level. HR, hazard ration; CI, confidence interval. *** *p* < 0.001, as compared to control.

RNA sequencing data from The Cancer Genome Atlas (TCGA) Glioblastoma dataset showed that the mRNA expression of *NOX4* was enriched in glioblastoma compared to low-grade glioma and non-tumor tissues (Figures 1A,B). Further, the Chinese Glioma Genome Atlas (CGGA) and TCGA Low-Grade Glioma datasets showed that *NOX4* expression was significantly higher in glioblastoma comparing with other histological types (Figures 1C,D). Consistent with these findings, immunohistochemistry staining and western blotting of clinical samples showed that the NOX4 protein level was higher in high-grade glioma than in low-grade glioma (Figures 1E,F). Supplementary Table S1 lists patient information. Regarding the prognostic value of NOX4 in glioma, the CGGA and TCGA Low-Grade Glioma datasets showed that patients with glioma with a higher *NOX4* expression had worse overall survival than those with a lower *NOX4* expression (Figures 1G,H). These results suggested that *NOX4* was upregulated and acted as a potential oncogene in glioblastoma.

### Correlation of *NOX4* with the prognosis of glioma in an intracranial xenograft model

**FIGURE 2:**
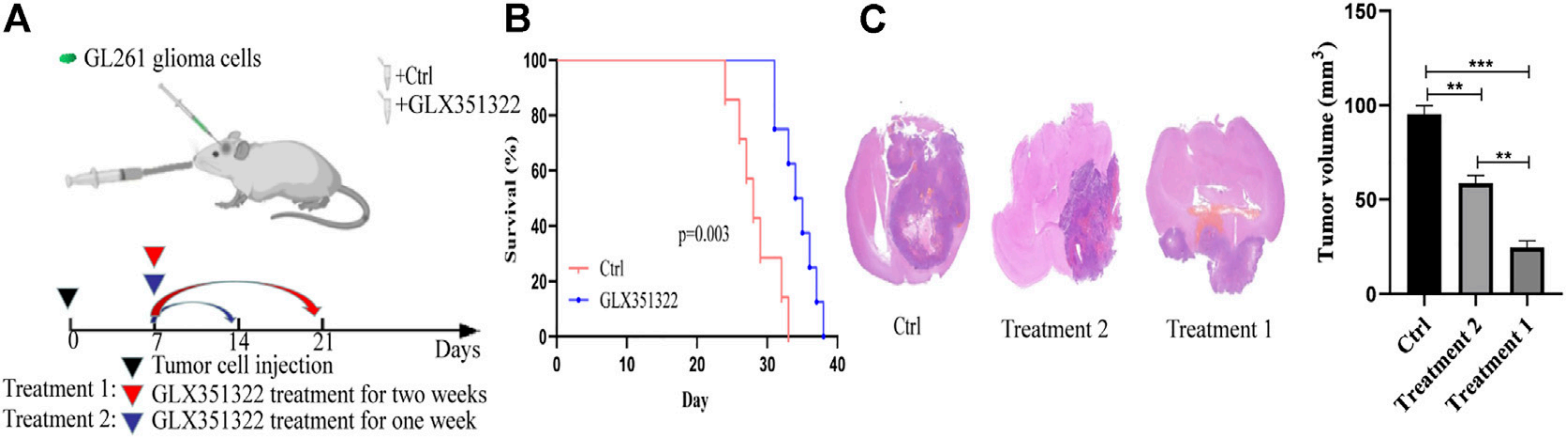
Knockdown of NOX4 significantly inhibits tumor growth *in vivo*. **(A)** Schematic diagram of targeted therapy *in vivo*. Xenograft models were established by intracranial injection of (1 × 10 ^6^ cells), which were directly inoculated into the lateral ventricle of nude mice. The GLX351322 or drinking water was treated every day after 1 week following the xenotransplantation, and treatment was repeated one or 2 weeks; **(B)** Kaplan–Meier survival curves of tumor-bearing mice received the indicated treatments (n = 7); **(C)** H&E staining of the brains isolated from mice received the indicated treatments. Scare bar: 100 µm.

Survival curves showed that mice in the control group died significantly earlier than those in treatment group 1 (Figure 2B). Hematoxylin–eosin staining of brain tissues of tumour-bearing mice showed a significantly greater average volume of glioma in the control group than in the treatment groups *in vivo* (Figure 2C). These results suggested that *NOX4* could promote glioma growth and progression, consistent with TCGA and CGGA dataset results.

### Induction of angiogenesis by *Nox4* in an ROS-dependent manner

**FIGURE 3:**
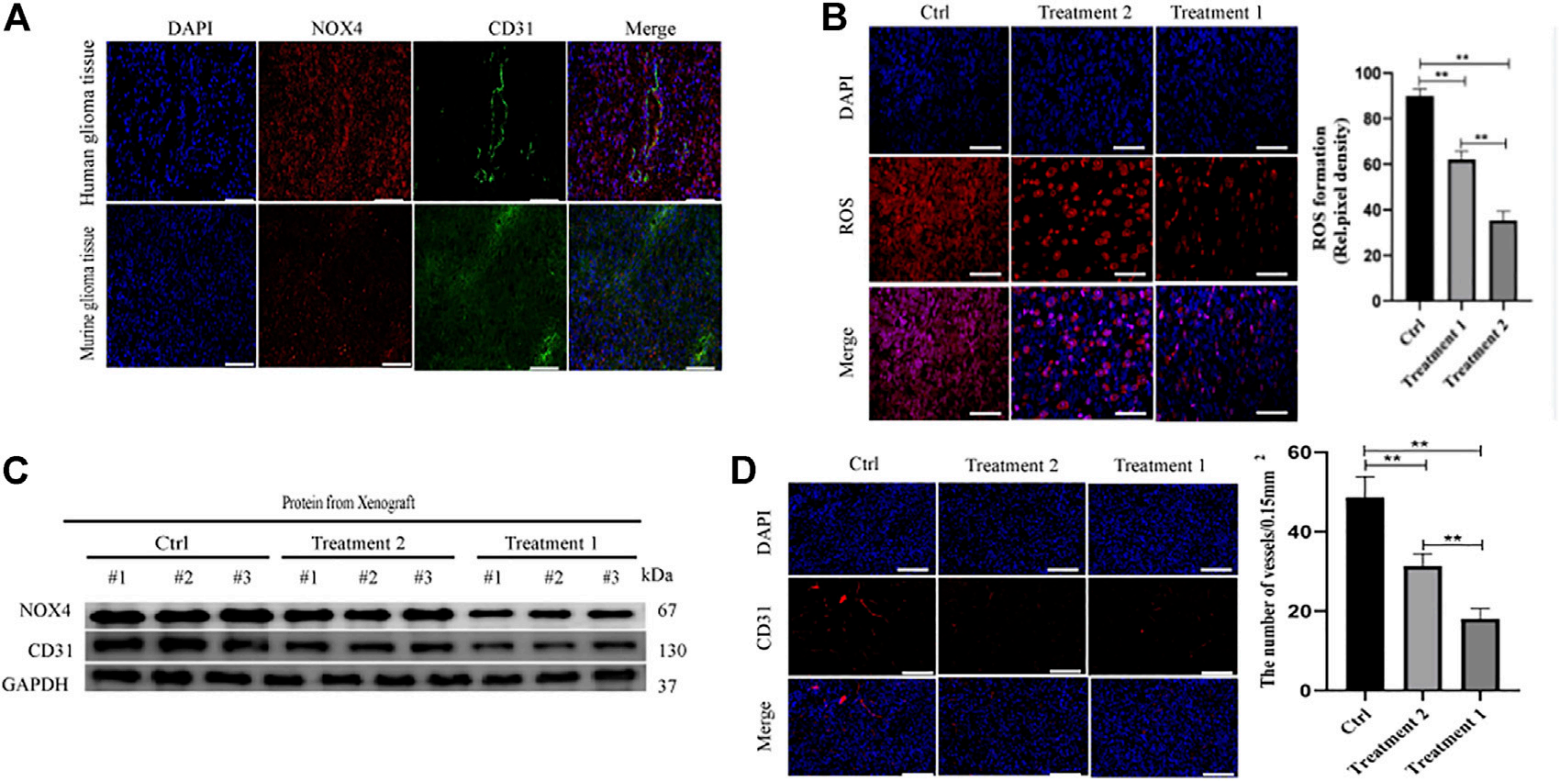
Nox4 induced angiogenesis in a ROS dependent manner **(A)** Both huamn and murine glioma tissues in an intracranial Xenograft model were co-stained by immunofluorescence for-anti-NOX4- (red), anti-CD31^−^ (green), showing NOX4 expression (red) in endothelia (green). Scale bars, 50 μm; **(B)** Representative images of ROS production. Scale bars, 50 μm; **(C)** Western blot analysis of the levels of the angiogenesis-associated protein (CD31) and NOX4 in glioma tissues; **(D)** Representative immunohistochemical analysis of CD31 expression in intracranial Xenograft tissue. Scale bars, 50 μm. Three independent experiments were performed. The data are presented as the means ± SEM. All images represented as the mean ± SD of three independent experiments; *p < 0.5; **p < 0.01; *** *p* < 0.001.

Human brain cell-type enrichment profiles (https://cell-enrichment.shinyapps.io/Brain/) have shown that *NOX4* is related to the glioblastoma-associated endothelium. Angiogenesis is common in glioblastoma and contributes to its aggressive behavior. We analyzed the correlation between angiogenesis and the *NOX4* expression in glioblastoma and then performed immunofluorescent co-staining against CD31. Clinical samples of glioblastoma showed *NOX4* expression in CD31-positive endothelial cells attached to the newly forming vasculature within the tumor region. Murine glioblastoma models confirmed this finding. *NOX4*-positive and CD31-positive endothelial cells showed a strong correlation (Figure 3A). These results suggested that *NOX4* may play a vital role in angiogenesis.

Our previous findings indicated that *NOX4* was mainly expressed in endothelial cells of glioblastoma, leading us to determine whether or not *NOX4* regulates angiogenesis in glioma. *Nox4* is a critical mediator of ROS production and tumor progression (Hsieh et al., 2012). To further determine the mechanism of *NOX4* involvement in glioma, we compared ROS levels between the control and LX351322 treatment groups.

Targeting *NOX4* with GLX351322 affect the ROS level (Figure 3B). We stained tissues in the control and GLX351322 treatment groups for the angiogenesis marker CD31 and performed western blotting (Figures 3C,D). The control group showed more abundant CD31^+^ blood vessels compared to the GLX351322 treatment group. These results indicated that *NOX4* induced angiogenesis in an ROS-dependent manner in endothelial cells of glioblastoma *in vivo*.

### Promotion of angiogenesis in glioblastoma by TSPO *in vivo*

TSPO expression in glioma is correlated with the grade of malignancy and survival. It occurs in microglia, tumor-associated macrophages, endothelial cells, and pericytes (Cai et al., 2020). We explored the role of TSPO in glioma by targeting it with the synthetic ligand XBD173.

**FIGURE 4:**
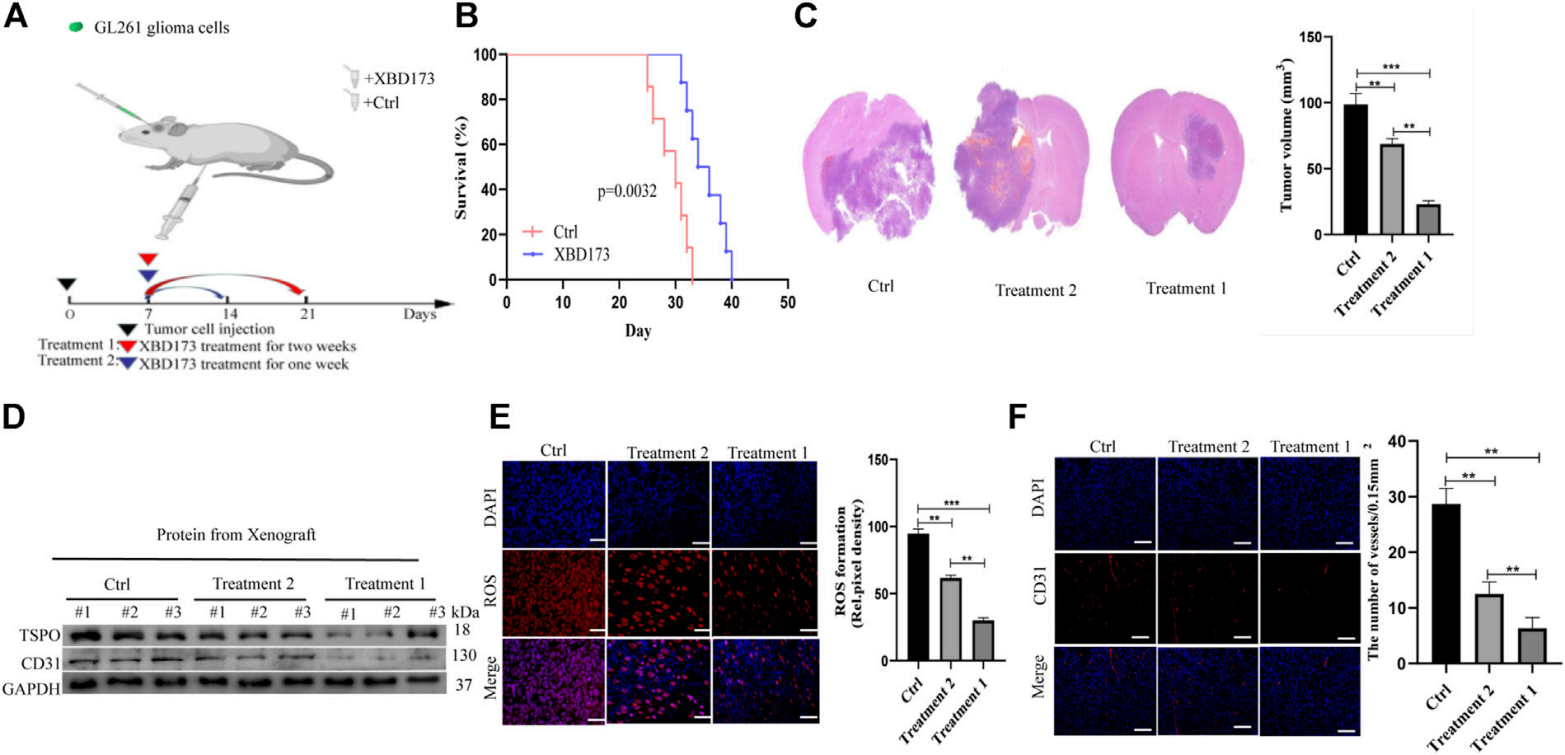
TSPO promotes GBM pathogenesis and angiogenesis *in vivo*. **(A)** Schematic diagram of targeted therapy *in vivo*. Xenograft models were established by intracranial injection of (1 × 10 ^6^ cells), which were directly inoculated into the lateral ventricle of nude mice. The XBD173 or DMSO was treated every day after 1 week following the xenotransplantation, and treatment was repeated one or 2 weeks; **(B)** Kaplan–Meier survival curves of tumor-bearing mice received the indicated treatments (n = 7); **(C)** H&E staining of the brains isolated from mice received the indicated treatments. Scare bar: 100 μm; **(D)** Western blot analysis of the levels of the angiogenesis-associated protein (CD31) and TSPO in glioma tissues; **(E)** Representative images of ROS production. Scale bars, 50 μm; **(F)** Representative immunohistochemical analysis of CD31 expression in intracranial Xenograft tissue. Scale bars, 50 μm. Three independent experiments were performed. The data are presented as the means ± SEM. All images represented as the mean ± SD of three independent experiments; *p < 0.5; **p < 0.01; *** *p* < 0.001.

We treated xenograft tumors of C57BL/6J mice with daily intraperitoneal injections of XBD173 for one or 2 weeks (Figure 4A). The XBD173 treatment group showed more survival benefit compared to the control group (Figure 4B). The tumor size was significantly smaller in the XBD173 treatment group compared to the control group (Figure 4C). These results indicated that TSPO could promote glioma growth and malignancy *in vivo*.

Further, we investigated the anti-angiogenic potential of XBD173. Western blotting showed more reduction of angiogenesis in the XBD173 treatment group than in the control group and successful knockdown of TSPO in the XBD173 treatment group (Figure 4D). Being a rich source of ROS, reactive mononuclear phagocytes are drivers of neurodegeneration. We analyzed if targeting TSPO with XBD173 affects ROS production of glioblastoma *in vivo*. The XBD173 treatment group showed lower ROS levels and number of vessels compared to the control group (Figures 4E,F). These results suggested that XBD173 attenuated both ROS production and angiogenesis.

### Trigger of ROS production by TSPO *via NOX4* in glioblastoma

We evaluated the molecular pathways involved in the role of TSPO in phagocyte ROS production. TSPO can regulate microglial functions in angiogenesis in an NOX1-dependent manner (Wolf et al., 2020). ROS is mainly produced by NOX and may be produced by mitochondria under certain conditions. We aimed to validate the hypothesis that TSPO regulates angiogenesis *via NOX4* in glioblastoma. We performed immunofluorescent co-staining of *NOX4* against Iba1.

**FIGURE 5:**
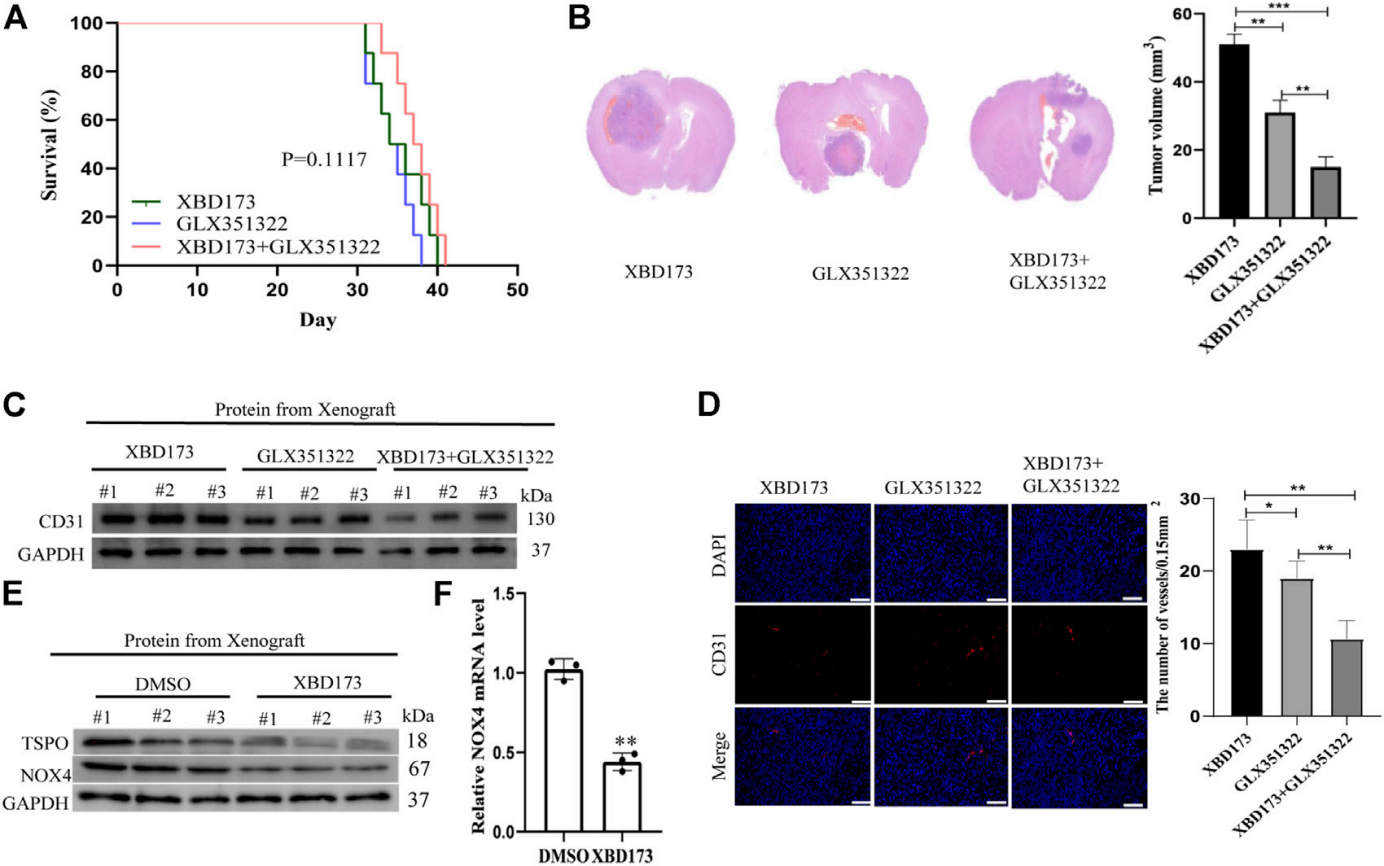
TSPO triggered ROS production *via* NOX4 in an intracranial xenograft glioma model. **(A)** Survival curves for the mouse intracranial xenograft glioma model following the control, 2 weeks injection of the XBD173 or GLX351322 treatment in normal saline; **(B)** H&E staining of the brains isolated from mice received the indicated treatments. Scare bar: 100 μm; **(C)** Western blot analysis of the levels of the angiogenesis-associated protein (CD31) in the 2 weeks injection of the XBD173 group, GLX351322 treatment in normal saline group and both the treatments groups; **(D)** Representative immunohistochemical analysis of CD31 expression in intracranial Xenograft tissue. Scale bars, 50 μm. Three independent experiments were performed. The data are presented as the means ± SEM. All images represented as the mean ± SD of three independent experiments; *p < 0.5; **p < 0.01; *** *p* < 0.001; **(E)** Western blot verified the loss of TSPO and NOX4 expression in an intracranial xenograft glioma model in which the gene was knocked down using the synthetic ligand XBD173; **(F)** The relative mRNA expression levels of NOX4 are compared between the DMSO and XBD173 group based on real-time PCR results; *p < 0.5; **p < 0.01; *** *p* < 0.001.

Clinical samples of glioblastoma showed *NOX4* expression in Iba1-positive microglia and macrophages (Supplementary Figure S1). We treated xenograft tumors of C57BL/6J mice with both the synthetic ligand XBD173 and GLX351322 and compared it to XBD173 or GLX351322 alone. The application of both XBD173 and GLX351322 did not yield survival benefit (Figure 5A). The average tumor size of the combined XBD173 and GLX351322 group was significantly smaller than that of the XBD173 or GLX351322 group (Figure 5B). Additionally, the ROS level and angiogenesis were reduced in the combined XBD173 and GLX351322 group compared to the XBD173 or GLX351322 group (Figures 5C–F). These results suggested that *NOX4* in endothelial cells plays a key role in ROS production in glioblastoma and critically depends on TSPO in microglia.

## Discussion

In recent decades, great progress has been made in tumor research. Glioma is a common malignant brain tumor characterized by abnormal angiogenesis and metabolism. In its pathological progression, metabolic abnormalities interact with angiogenesis (Krishna et al., 2021). Cellular metabolism can produce and accumulate abundant extracellular ROS (Li et al., 2020). Nevertheless, aerobic cells and tissues are imbalanced because of excessive accumulation of extracellular ROS, or “oxidative stress,” which is associated with tumor progression (Miller, 2009). In many pathophysiological processes, both extracellular and intracellular ROS are related to angiogenesis (Siciliano et al., 2020). Intracellular ROS plays an important role *via* vascular endothelial growth factor (VEGF) signaling in endothelial cells (Zheng et al., 2021). The NOX family is a plasma membrane-binding enzyme that produces superoxides and is the main source of ROS in the tumor microenvironment (Ohgaki and Kleihues, 2013).

In this study, *NOX4* expression was analyzed in glioma samples from patients and mouse models. The results indicated that *NOX4* is mainly distributed in endothelial cells and can affect angiogenesis in glioma. Furthermore, we investigated the effect of *NOX4* on angiogenesis in glioma and progression in an *in vivo* tumor model using the *NOX4* inhibitor GLX351322. The NOX family, which includes NOX1, NOX2, NOX3, NOX4, and NOX5 subtypes, is a key mediator of cyclic hypoxia-mediated ROS production and tumor progression (Cicone et al., 2019). Excessive ROS production by NOXs leads to oxidative stress and tumor progression (Zhai et al., 2020). NOXs perform multiple functions in tumors, such as proliferation, angiogenesis, anti-apoptosis, epithelial–mesenchymal transition, glycolysis, and metastasis (Lamantia et al., 2014; Lakshmanachetty et al., 2021). They may be closely related to the inflammatory tumor microenvironment, in which oxidants act as positive signaling mediators for tumor growth (Guadagno et al., 2018). Nevertheless, the relationship between the tumor microenvironment and NOXs remains unclear. In our study, ROS production in glioma was strongly regulated by *Nox4*.

TSPO is a powerful and precise biomarker of microglial activation in neurodegenerative diseases (Ding et al., 2014). It is elevated on PET in Alzheimer’s disease, mild cognitive impairment (Iwata et al., 2020), and almost all types of central nervous system pathologies and reduced in some psychiatric disorders, such as schizophrenia (Rossi et al., 1988). It is expressed in tumor cells and microenvironments, particularly in tumor-associated myeloid cells, pericytes, and endothelial cells. Its expression in glioblastoma tumor tissue or possible mechanism remains unclear. In addition, PET using the TSPO radiation ligand is not a standard imaging modality for patients with brain tumors (Zhang et al., 2020; Yang T. et al., 2021; Holzl et al., 2021). The relationship between CD31^+^ and TSPO^+^ endothelial cells is found in blood vessels of different sizes, including capillaries (Huang et al., 2019). The presence of TSPO^+^ blood vessels in the brain of any disease suggests that endothelial TSPO may be constructive (Duan et al., 2019). This finding is associated with the binding and distribution of TSPO ligands in the brain parenchyma (Duan et al., 2019). In this study, targeting the specific ligand XBD173 showed that the TSPO signaling pathway played a key role in reducing angiogenesis in glioblastoma, consistent with the main pathological features of neovascular age-related macular degeneration. We hypothesized that TSPO plays a critical role in *NOX4*-mediated extracellular ROS production in glioblastoma.

A TSPO-dependent regulation of NOX enzymes has been proposed (Wierstra et al., 2019; Yang Z. et al., 2021). Microglia produce extracellular ROS *via* NOX1. TSPO is involved in the production of mitochondrial ROS in relation to cell signal transduction (Venkatesh et al., 2019). Therefore, we investigated the role of TSPO in subsequent *NOX4*-derived ROS generation in glioblastoma. ROS was significantly reduced in TSPO-deficient glioblastoma. *NOX2* is the main source of ROS in phagocytes (Hamerlik et al., 2012); however, *NOX1*-and *NOX4*-dependent ROS productions may occur in microglia of different neurological diseases (Acharya et al., 2016; Blanchart et al., 2017). Although only a few *in vivo* studies have investigated the role of *NOX2* or *NOX4* in deficient mice (Hamerlik et al., 2012; Feng et al., 2018), most studies on microglia have been conducted using cell lines. In this study, TSPO-mediated angiogenesis was blocked by XBD173, which inhibited *Nox4* expression and the production of acute *NOX4*-derived ROS after stimulation. Therefore, these signal cascades in angiogenesis should be identified in future studies. In addition, botanical drugs targeting TSPO-NOX4 axic have shown great potential in the treatment of glioblastoma, such as paeoniflorin, which will be further studied in the later stage.

This study has some limitations. First, targeting or knocking-out TSPO by synthesizing the ligand XBD173 confirmed that TSPO could inhibit phagocytic reactivity in the laser-induced mouse neovascular age-related macular degeneration model. However, we only analyzed the effect of TSPO using XBD173. The synthetic ligand XBD173 is a fat-soluble reagent and, therefore, easily crosses the blood–brain barrier, thus becoming a potential substitute of brain tumor chemotherapy in clinical practice. Therefore, the exact composition and function of these TSPO proteins in glioma should be further explored. Second, GLX351322 knocked-out and chemically inhibited the NOX4 enzyme. Although often referred to as specific *NOX4* inhibitors, these substances exhibit many undergo side reactions with questionable specificity (Radoul et al., 2021). Further studies are required in this direction. In addition, XBD173 reduces the expression of angiogenic factors and choroidal neovascularization by regulating phagocytes. Although Ang1, Ang2, and Vegf were expressed by phagocytes *in vivo*, only VEGF was generated and secreted by stimulated microglia. The specific interactions between phagocytes and endothelial cells and other related signaling pathways that may play a role in angiogenesis in glioma should be further investigated.

## Conclusion

**FIGURE 6:**
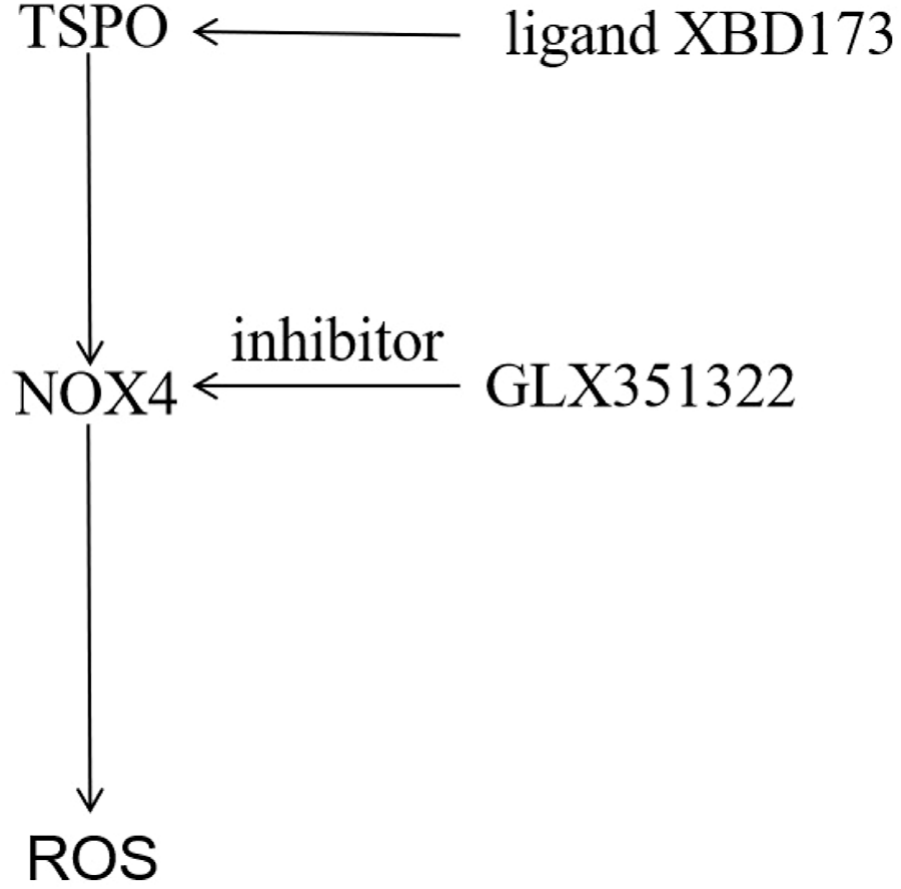
Mechanistic model of the malignant phenotype mechanism of GBM mediated by TSPO. The TSPO was the upstream target of NOX4-derived MitoROS, which was necessary for NOX4-derived MitoROS-induced angiogenesis in GBM.

In this study, both TSPO and *NOX4* promoted angiogenesis in glioblastoma. This was the first study to demonstrate that TSPO was the upstream target of *NOX4*-derived mitochondrial ROS, necessary for NOX4-derived mitochondrial ROS-induced angiogenesis in glioblastoma (Figure 6). TSPO–*Nox4* signaling seems to be a promising molecular therapeutic target for glioblastoma.

## Data Availability

The raw data supporting the conclusions of this article will be made available by the authors, without undue reservation.
