# Supplementary figures and images for: Role of the TSPO–NOX4 axis in angiogenesis in glioblastoma
Source: Front Pharmacol. 2022 Oct 7;13:1001588. doi: 10.3389/fphar.2022.1001588 (PMC9585329; doi:10.3389/fphar.2022.1001588)

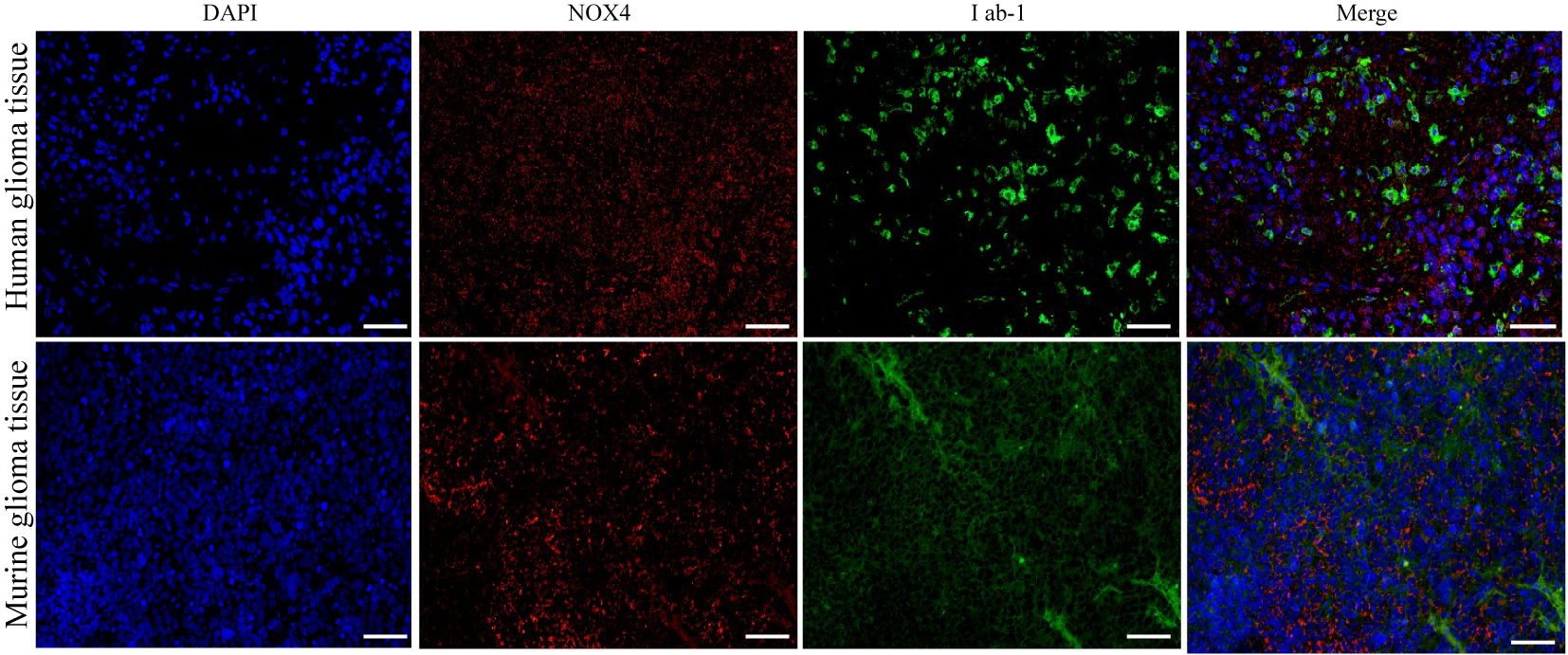

Supplement: Supplementary file 2 [file Image1.tif]
